# Nodular Vasculitis Following Aromatase Inhibitor Therapy for Breast Cancer: A Case Report

**DOI:** 10.7759/cureus.108680

**Published:** 2026-05-11

**Authors:** Samuel Srinivasan, Maria Jamil, Sarine Tahmazian, Javairia Jamil, Hiba Jabbour-Aida

**Affiliations:** 1 Internal Medicine, Henry Ford Health System, Detroit, USA; 2 Internal Medicine, Gulf Medical University, Ajman, ARE; 3 Hematology and Medical Oncology, Henry Ford Health System, Detroit, USA

**Keywords:** aromatase inhibitor therapy, breast cancer, breast cancer treatment, erythema induratum, nodular vasculitis

## Abstract

Letrozole is a potent and generally well-tolerated aromatase inhibitor that blocks endogenous estrogen production in patients with hormone receptor-positive breast cancer. Although letrozole has a known profile of mild side effects, cutaneous manifestations associated with this therapy are rare. Here, we describe a patient who had undergone definitive treatment for hormone receptor-positive breast cancer and developed nodular vasculitis several months after initiation of letrozole. The patient presented with painful, hyperpigmented subcutaneous nodules along the bilateral thighs and shins. Histopathology revealed septal and lobular panniculitis with neutrophilic and granulomatous inflammation. Symptom resolution following discontinuation of letrozole and symptom recurrence after drug reinitiation supported a final diagnosis of erythema induratum. This case highlights a rare but clinically significant cutaneous adverse effect associated with letrozole therapy, underscoring the importance of recognizing drug-induced nodular vasculitis and emphasizing the need to balance symptom management with ongoing cancer therapy.

## Introduction

Aromatase inhibitors (AIs) represent a cornerstone of endocrine therapy for hormone receptor-positive breast cancer in postmenopausal women [1]. These agents inhibit the aromatase enzyme responsible for peripheral conversion of androgens to estrogens, thereby markedly reducing circulating estrogen levels and limiting estrogen-driven tumor growth [1,2]. Letrozole, a potent third-generation AI, has demonstrated substantial efficacy in both adjuvant and advanced disease settings, significantly reducing recurrence risk and improving disease-free survival [2].

The safety profile of letrozole is well established. Common adverse effects include hot flashes, arthralgia, myalgia, fatigue, and reduced bone mineral density [2]. Cutaneous adverse events are relatively uncommon but increasingly reported, including vasculitic eruptions, erythema nodosum, and autoimmune dermatoses [3-5]. As the duration and prevalence of AI therapy increase, recognition of rare inflammatory skin reactions has become increasingly important.

Nodular vasculitis, historically referred to as erythema induratum of Bazin (overlapping entities used interchangeably in this article), is a chronic inflammatory disorder of the subcutaneous fat that predominantly affects women and typically presents as tender nodules on the lower extremities [4,6]. Histopathologically, it is characterized by predominantly lobular panniculitis with granulomatous inflammation, fat necrosis, and associated vasculitis involving small- to medium-sized vessels [7]. Although originally associated with tuberculosis infection, noninfectious etiologies, including autoimmune conditions, malignancy, and drug-induced hypersensitivity reactions, are now well recognized [7,8]. Here, we present a novel case of letrozole-associated nodular vasculitis/erythema induratum, a finding not currently in existing literature, contributing to the expanding spectrum of aromatase inhibitor-related cutaneous toxicities.

## Case presentation

A 57-year-old postmenopausal woman with a history of estrogen receptor- and progesterone receptor-positive breast cancer presented to the dermatology clinic with a several weeks' history of painful subcutaneous nodules involving both lower extremities. Her oncologic history was significant for a definitive bilateral mastectomy followed by re-excision for persistently positive surgical margins. She had been initiated on adjuvant endocrine therapy with letrozole approximately four months prior to the onset of her dermatologic symptoms.

**Figure 1:**
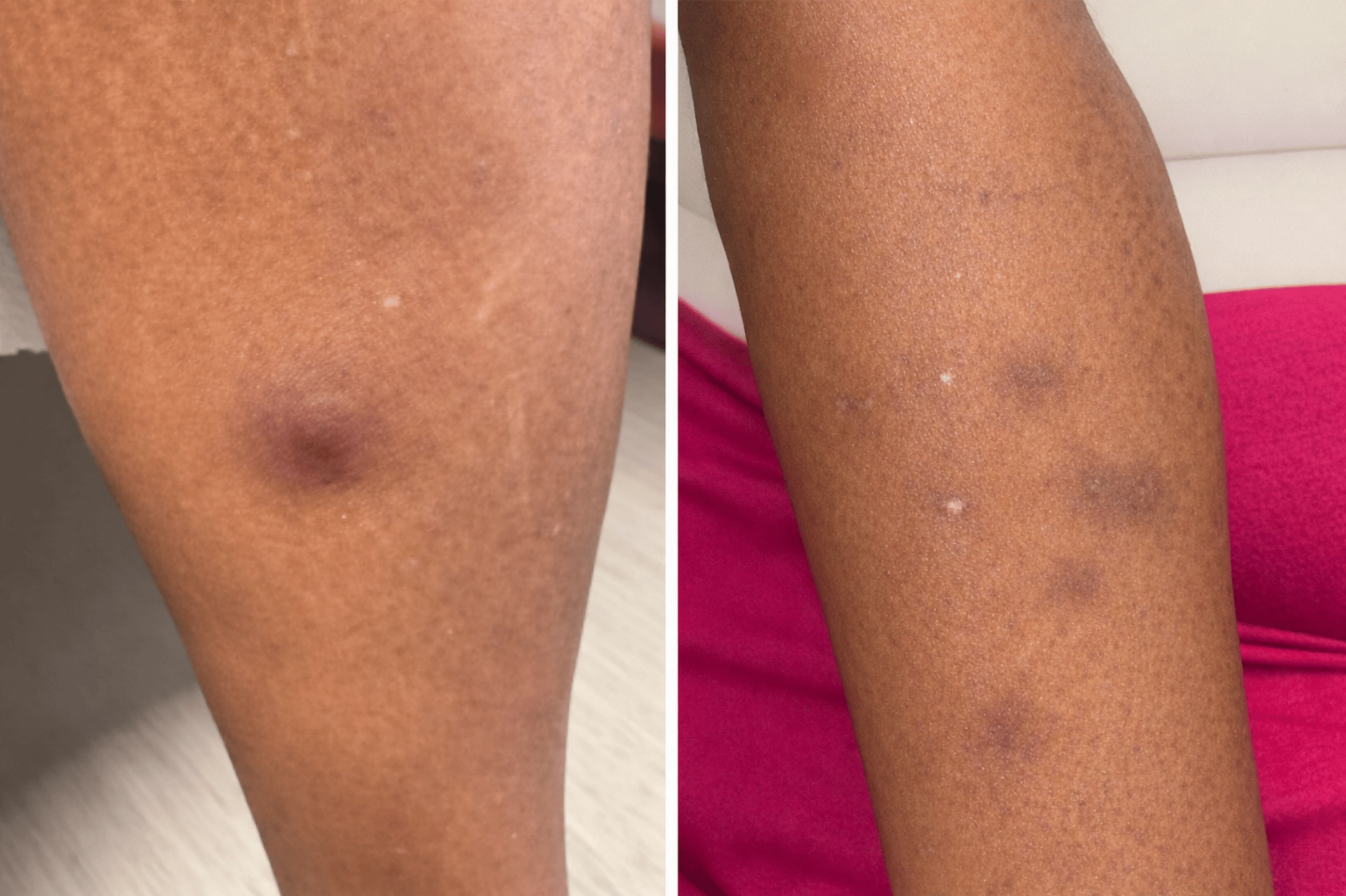
Clinical presentation of nodular vasculitis involving the lower extremities. Left: Solitary hyperpigmented, violaceous subcutaneous nodule on the anterior lower leg. Right: Multiple hyperpigmented subcutaneous nodules distributed along the lower extremity.

The patient denied recent infections, constitutional symptoms, trauma, or new medications aside from letrozole. She reported no fevers, weight loss, cough, arthralgias, or systemic complaints. Her past medical history was otherwise unremarkable, and she was not immunocompromised. Physical examination revealed multiple tender, hyperpigmented subcutaneous nodules distributed along the bilateral anterior thighs and shins (Figure 1). The lesions were firm, poorly demarcated, and non-ulcerated.

**Figure 2:**
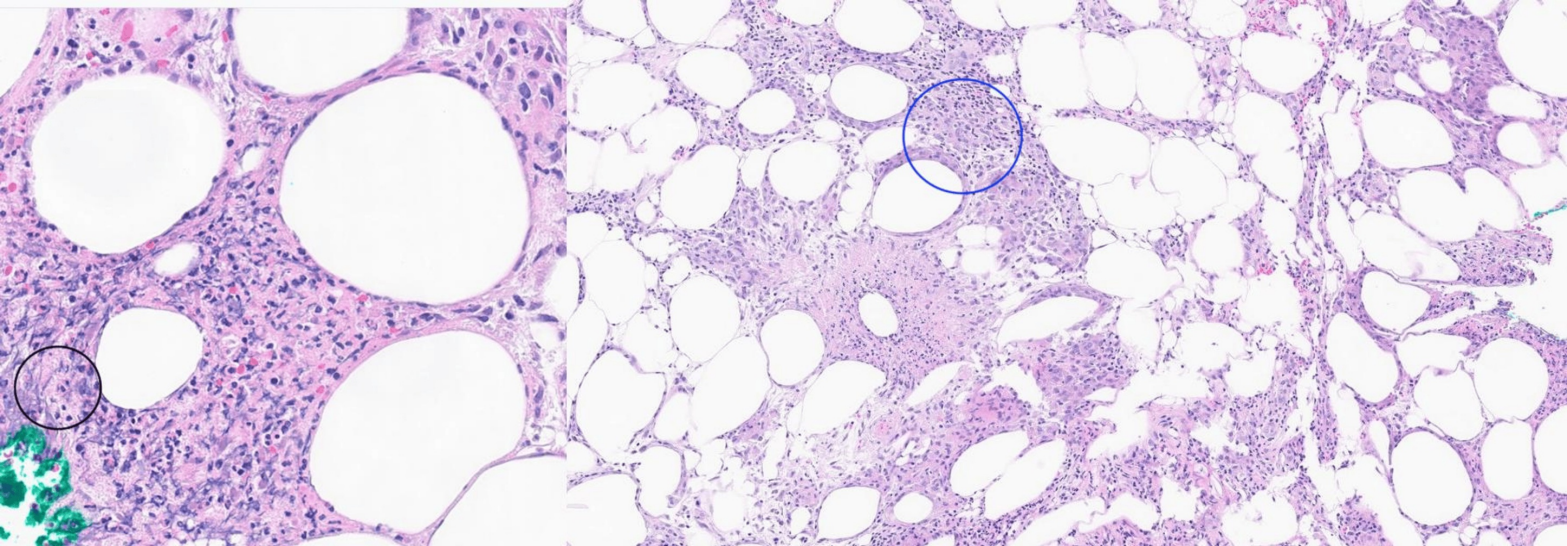
Biopsy revealing lobular panniculitis with neutrophilic and granulomatous inflammation; focal necrosis was present as well. Left image: Black circle demonstrating loss of cellular architecture and increased eosinophilia demonstrating focal necrosis. Right image: Blue circle demonstrating mixed septal and lobular inflammation with neutrophilic and granulomatous inflammation.

A punch biopsy was obtained from a representative lesion on the lower extremity. Histopathologic examination demonstrated mixed septal and lobular panniculitis with neutrophilic and granulomatous inflammation (Figure 2). Associated vasculitic changes were noted. Special stains and microbiologic evaluation did not demonstrate infectious organisms. The differential diagnosis included nodular vasculitis/erythema induratum, infective panniculitis, and systemic vasculitis such as granulomatosis with polyangiitis. Erythema nodosum was considered less likely given the presence of prominent lobular inflammation.

Approximately two months after initial presentation, letrozole therapy was discontinued to assess for potential drug-induced etiology. At follow-up one month after drug cessation, the patient reported improvement in pain and erythema, and no new lesions were observed. The clinical course favored a diagnosis of erythema induratum. She was initiated on potassium iodide for symptomatic relief.

After approximately two weeks of potassium iodide therapy, the patient resumed letrozole as part of her ongoing adjuvant cancer treatment and was also started on ribociclib. Approximately 2.5 months after reinitiation of letrozole, she developed a new mildly painful nodule on the left lower leg. This recurrence following drug rechallenge further supported a diagnosis of letrozole-associated nodular vasculitis. The new lesion was treated with intralesional triamcinolone acetonide, while potassium iodide therapy was continued. Letrozole therapy was maintained after shared decision-making given the patient’s preference to prioritize oncologic benefit.

## Discussion

The growing complexity of systemic oncologic therapies has been paralleled by an expanding spectrum of treatment-related adverse events. Although aromatase inhibitors are generally considered well tolerated, dermatologic toxicities, while uncommon, can significantly impact patients' quality of life and adherence to therapy [3]. Reported cutaneous reactions to aromatase inhibitors include vasculitis, erythema nodosum, subacute cutaneous lupus erythematosus, and other inflammatory dermatoses [4,5]. Nodular vasculitis associated with letrozole, however, remains exceedingly rare.

Erythema induratum is a form of nodular vasculitis that typically presents as recurrent tender nodules involving the lower legs of women [6,7]. Histologically, it demonstrates predominantly lobular panniculitis with granulomatous inflammation and vasculitic changes, often accompanied by fat necrosis [7,8]. While tuberculosis remains the classical cause, most modern cases in developed countries are non-tuberculous and may be idiopathic, autoimmune-related, or drug-induced [7,8].

Drug-induced panniculitis is uncommon but documented with several medications, supporting a delayed hypersensitivity-mediated mechanism [9,10]. In our patient, multiple factors strongly support letrozole-induced nodular vasculitis. There was a clear temporal association between initiation of letrozole and symptom onset. Partial clinical improvement followed drug discontinuation (dechallenge), and recurrence of lesions occurred upon re-exposure (rechallenge). Rechallenge positivity is considered one of the strongest indicators of causality in adverse drug reactions. Application of the Naranjo Adverse Drug Reaction Probability Scale would classify this reaction as “probable” (score of 7) based on temporal sequence, dechallenge response, and rechallenge recurrence [11].

The exact pathophysiologic mechanism linking aromatase inhibition to panniculitis remains unclear. Estrogen exerts significant immunomodulatory effects, influencing cytokine expression, T-cell activation, and inflammatory signaling pathways [12]. Estrogen deprivation induced by aromatase inhibitors may alter immune homeostasis and predispose susceptible individuals to inflammatory or hypersensitivity-mediated reactions within subcutaneous adipose tissue. Although speculative, this biological plausibility strengthens the observed association.

Alternative etiologies were carefully considered. Infectious panniculitis was unlikely given the absence of systemic symptoms, immunocompromise, or microbiologic evidence of infection [10]. Additionally, the patient demonstrated no features suggestive of systemic vasculitis, such as granulomatosis with polyangiitis. The localized clinical presentation and temporal drug relationship favored a drug-induced inflammatory process.

Management of drug-induced panniculitis in oncology presents a therapeutic challenge. Aromatase inhibitors significantly reduce recurrence risk in hormone receptor-positive breast cancer [1,2], and discontinuation may compromise long-term outcomes. Therefore, management requires individualized, multidisciplinary decision-making. In this case, shared decision-making led to continuation of letrozole therapy with adjunctive potassium iodide and intralesional corticosteroid treatment, prioritizing oncologic benefit while mitigating dermatologic symptoms.

This case broadens the spectrum of reported aromatase inhibitor-associated inflammatory dermatoses and underscores the importance of recognizing rare but clinically significant cutaneous toxicities. Written informed consent was obtained from the patient for the publication of this case report and associated clinical images. The patient was informed that identifying information would be kept confidential and that all reasonable efforts would be made to ensure anonymity.

## Conclusions

Aromatase inhibitors, including letrozole, are a mainstay of breast cancer therapy for hormone receptor-positive breast cancer. Although these drugs are generally well-tolerated with favorable side effect profiles, here we described a rare cutaneous manifestation of letrozole-associated erythema induratum. This report expands the spectrum of possible adverse effects associated with letrozole therapy. Balancing the need for symptom control with the risk of disease recurrence is a common yet challenging dilemma in oncology, and awareness of nodular vasculitis as a potential adverse effect is essential when evaluating inflammatory cutaneous manifestations in patients on aromatase inhibitors.
